# Ecological Connectivity of *Trypanosoma cruzi* Reservoirs and *Triatoma pallidipennis* Hosts in an Anthropogenic Landscape with Endemic Chagas Disease

**DOI:** 10.1371/journal.pone.0046013

**Published:** 2012-09-26

**Authors:** Janine M. Ramsey, Ana E. Gutiérrez-Cabrera, Liliana Salgado-Ramírez, A. Townsend Peterson, Victor Sánchez-Cordero, Carlos N. Ibarra-Cerdeña

**Affiliations:** 1 Centro Regional de Investigación en Salud Pública, Instituto Nacional de Salud Pública, Tapachula, Chiapas, México; 2 Biodiversity Institute, The University of Kansas, Lawrence, Kansas, United States of America; 3 Instituto de Biología, Universidad Nacional Autónoma de México, Ciudad Universitaria, Distrito Federal, México; Instituto Butantan, Laboratório Especial de Toxinologia Aplicada, Brazil

## Abstract

Traditional methods for Chagas disease prevention are targeted at domestic vector reduction, as well as control of transfusion and maternal-fetal transmission. Population connectivity of *Trypanosoma cruzi*-infected vectors and hosts, among sylvatic, ecotone and domestic habitats could jeopardize targeted efforts to reduce human exposure. This connectivity was evaluated in a Mexican community with reports of high vector infestation, human infection, and Chagas disease, surrounded by agricultural and natural areas. We surveyed bats, rodents, and triatomines in dry and rainy seasons in three adjacent habitats (domestic, ecotone, sylvatic), and measured *T. cruzi* prevalence, and host feeding sources of triatomines. Of 12 bat and 7 rodent species, no bat tested positive for *T. cruzi*, but all rodent species tested positive in at least one season or habitat. Highest *T. cruzi* infection prevalence was found in the rodents, *Baiomys musculus* and *Neotoma mexicana*. In general, parasite prevalence was not related to habitat or season, although the sylvatic habitat had higher infection prevalence than by chance, during the dry season. Wild and domestic mammals were identified as bloodmeals of *T. pallidipennis*, with 9% of individuals having mixed human (4.8% single human) and other mammal species in bloodmeals, especially in the dry season; these vectors tested >50% positive for *T. cruzi*. Overall, ecological connectivity is broad across this matrix, based on high rodent community similarity, vector and *T. cruzi* presence. Cost-effective *T. cruzi*, vector control strategies and Chagas disease transmission prevention will need to consider continuous potential for parasite movement over the entire landscape. This study provides clear evidence that these strategies will need to include reservoir/host species in at least ecotones, in addition to domestic habitats.

## Introduction

Chagas disease, caused principally by the vector transmission of *Trypanosoma cruzi*, is widespread and endemic in Latin America from southern United States to Argentina, with emerging non-vector transmission in other North American [1] and European countries [2]. Since 85% to 96% of *Trypanosoma cruzi* transmission to humans occurs via contact with infected feces from insects of the subfamily Triatominae (Reduviidae: Hemiptera), control programs primarily target vector densities in human abodes, with complementary transfusion, transplant, and congenital transmission control strategies [3]. Specific targets of vector control programs are domestic triatomine populations, since house infestation is the principal risk factor associated with *T. cruzi* infection in both rural and urban human populations.

Synanthropic adaptation of sylvatic triatomine populations occurs along a gradient of diversity reduction in mammal host communities, where a parallel filter favoring generalist/oportunistic host species exists. *Triatoma infestans*, at least formerly the primary vector responsible for Chagas disease transmission in many South American countries, is almost entirely domestic over its distribution, genetically isolated from sylvatic populations that persist only in a few regions [4]–[6]. The only other example of domestic triatomine population isolation is *Rhodnius prolixus* in Central America and Mexico, where the species was not native, and has not been capable of sylvatic habitat reinvasion [7]. Most triatomine species maintain gene flow between domestic, ecotone and/or sylvatic populations [8], [9], as in Mexico for all epidemiologically relevant species in the *phyllosoma* [10], *rubida* [11] and *dimidiata* species complexes [12]. *Triatoma pallidipennis* has been incriminated as one of the most important vector species for human *T. cruzi* infections in Mexico [13].

The first village-wide triatomine control trials using residual insecticides in Mexico was conducted in the town of Chalcatzingo, Morelos, which has high Chagas disease human case prevalence (2% in population under 15 yrs old) and vector infestation [14], [15], [16]. The intervention reduced *T. pallidipennis* significantly and eliminated *Triatoma barberi* domestic populations; however, *T. pallidipennis* re-infestation of domiciles was observed as early as six months post-intervention. At three years post-intervention, re-infestation of housing in Chalcatzingo exceeded 85% of original infestation levels, returning to pre-control levels of 60% by five years post intervention (Ramsey, personal communication). These results suggested that the habitat types and their connectivity could play an important role in the recovery of local vector and pathogen populations. Population continuity of disturbance-tolerant sylvatic mammal species and vector populations among habitats [17] may represent a positive and important mechanism for *T. cruzi* dispersal. Thus, evidence for habitat connectivity could influence choice of triatomine control strategies by providing essential information for more effective long-term integrated vector management. Many rural and urban settlements in Latin America have landscapes like those in Mexico, with human domiciles in a matrix of agricultural habitats and sylvatic fragments. Selection of cost-effective transmission control strategies should consider the degree of ecological connectivity and patterns of biotic interactions both within and between habitats of vector and host communities.

In Mexico, the most common landscape surrounding settlements infested with *dimidiata* or *phyllosoma* complex species is tropical deciduous forest [18]. Since deciduousness is a key ecological characteristic of seasonal forest [19], [20], major changes occur seasonally in animal community population patterns (reproduction, foraging, and dispersal) in response to the vegetation phenology cycle [21]. Therefore, the complete *T. cruzi* transmission system, including human Chagas disease transmission risk, also depends on seasonal synchronization of reservoir, host, and vector populations.

In the present study, we characterize and quantify the magnitude of ecological connectivity from a landscape perspective, using mammal communities and *T. cruzi* vertebrate-invertebrate interactions in an active Chagas disease transmission area. This analytical framework provides an opportunity to understand better the spatio-temporal dynamics of the primary ecological risk factors associated with human exposure to *T. cruzi*.

## Materials and Methods

### Ethics Statement

All necessary permits were obtained for the described field studies. All animal work was conducted under international and national guidelines with corresponding Biosecurity and Ethics approval from the National Institute of Public Health 50-64614. Verbal consent from the inhabitants of Chalcatzingo was obtained through community assemblies for domestic and peri-community collections. The field studies did not involve endangered or protected species.

### Study area

This study was conducted in and around the town of Chalcatzingo, Morelos, in central Mexico (elevation 1365 m, 18°43′22″N, 98°42′39″W) [16]. Annual mean precipitation is 900 mm, with <100 mm during the dry season and a rainy season extending from June to November; mean annual temperature averages 21.4°C (range 14.6–26.5°C).

**Figure 1:**
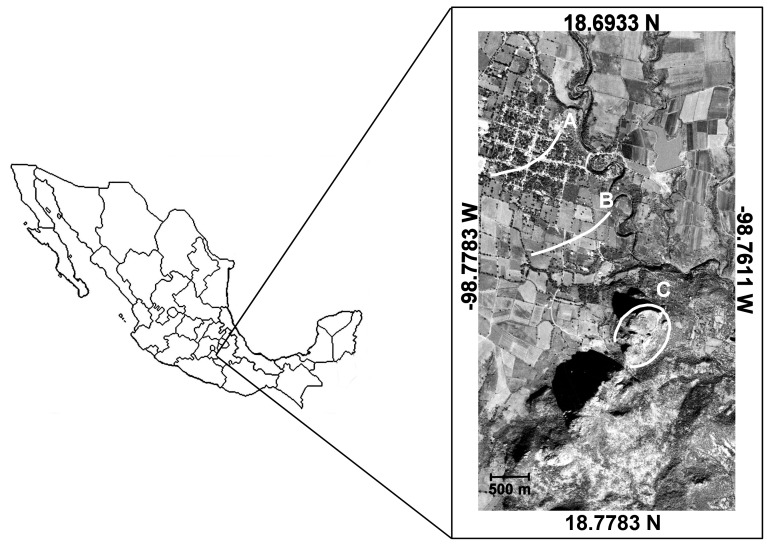
Orthophoto of the Chalcatzingo, Morelos, Mexico, landscape and sample transects (white lines) in each habitat: (A) domestic, (B) ecotone (crop areas), and (C) sylvatic. Landscape classification is qualitative given differences among the habitats: in the domestic area only houses and their corresponding gardens are available with 13% of households maintaining farm animals; in the ecotone, only crops, animal corrals or grazing areas are available, and in the sylvatic area, no housing, crops or grazing areas are not present, and low deciduous forest principally conserved.

Small mammals and triatomines were collected along three parallel linear transects located within each of three habitats, together constituting a single landscape (Figure 1): (A) the town which has a total of 650 private homes and 11 public buildings, as described elsewhere “domestic” [16], (B) a crop-growing belt surrounding the town, intermediate between the sylvatic forest and domestic habitats, planted with maize, beans, and sorghum “ecotone”, and (C) relatively conserved tropical forest deciduous habitat and thorn scrub, with the most common plant genera being *Acacia*, *Mimosa*, and *Pithecellobium*, covering rocky hills and shallow canyons “sylvatic”. Distances between transects A and B, and B and C were similar (1.5 km), while that from A to C was 3 km.

### Animal surveys

Small mammals (bats and rodents) and triatomines were collected from each habitat during the 2005 rainy season (September–October) and the 2006 dry season (March–April). Bats were collected between 20:00 and 01:00 hrs using three mist nets (6 m wide by 3 m high) set across paths and water causeways along each of the transects shown in Figure 1, for five consecutive nights in each habitat [22]. Additionally, in each season, a mist net was placed for 3 nights at the entrance of a cave in the sylvatic habitat.

Rodents were sampled only along terrestrial transects, since preliminary collections indicated no arboreal species at sylvatic sites (Fig. 1). One hundred Sherman traps were set 10 m apart for 20 consecutive days, for a total of 2000 trap-nights/habitat. Traps were baited with a mixture of corn and vanilla, which had previously proven to be a consistent and effective attractant for rodents.

Mammals were collected following the guidelines of the Mexican Secretary of Environment and Natural Resources [23] using a collection permit to VSC.

All animals collected were identified to species, weighed, measured for standard mammal museum measurements, sexed, and aged. To measure *T. cruzi* mammal infection rates, all collected animals were identified to species, anaesthetized, measured, weighed, and peripheral blood and cardiac tissue extracted and preserved immediately by drying or in ethanol, for posterior molecular *T. cruzi* identification; the complete animal was preserved in 70% EtOH. Preserved specimens were deposited as vouchers in the Centro Regional de Investigacion en Salud Pública/INSP, Instituto de Biologia, Universidad Nacional Autonoma de México, and Colegio de la Frontera Sur/CONACyT.

Triatomines were collected from sylvatic and ecotone habitats using canvas white light traps (2 m×3 m) for 4 hr every 5 nights, totaling 120 m^2^/season of light trapping. In domestic habitats, bugs were collected using 40-min timed searches inside and outside of 25 houses in the rainy season; in the dry season, collections were conducted in 10 houses for 30 min/house. All bugs collected were transported live weekly to the laboratory for *T. cruzi* infection detection, using direct observation of fecal samples and light microscopy. All bug specimens were subsequently preserved whole in 70% EtOH prior to midgut extraction and molecular analyses using PCR, for bloodmeal origin and *T. cruzi* infection.

### PCR for *T. cruzi* presence and bloodmeal identification

Cardiac tissue was extracted from anesthetized bat and rodent specimens according to standard procedures, and immediately cut into fine tissue blocks (2×2 mm^2^) and immersed in 70% ETOH. Genomic DNA from all tissue and gut samples was isolated using DNAzol (Invitrogen, San Diego, California, USA), following manufacturer's product instructions. The extracted DNA pellet was resuspended in 100 µL of 8 mM NaOH, and maintained at −20°C prior to final extraction and electrophoresis.

Presence of *T. cruzi* was confirmed from mammal tissue and triatomine midgut by amplifying conserved regions of the kinetoplast minicircle, using the oligonucleotide primers S34 5′-ACA CCA ACC CCA ATC GAA CC-3′ and S67 5′-TGG TTT TGG GAG GGG SSK TC-3′ [24]. A volume of 1 µL (representing 10–20 ng) of the total 100 µL DNA sample was added, for a final volume of either 12 µL or 25 µL, with 1× Master Mix (Taq polymerase in a 200 mM buffer at pH 8.5 of each dNTP and 1.5 mM MgCl_2_; Promega Corporation, Madison, Wisconsin, USA), and 0.5 µM of each primer, resulting in a 120 bp product. Initial DNA denaturalization was carried out at 94°C for 4 min, followed by 35 cycles of DNA denaturalization (94°C for 30 sec), oligo alignment (60°C for 30 sec), and chain elongation (72°C for 30 sec), ending with a final elongation period at 72°C for 10 min [25]. PCR products were separated and visualized on either 1% or 2% agarose gels stained with ethidium bromide and observed under UV light. A *T. cruzi* sample isolated from *T. pallidipennis* collected from the town of Temixco, Morelos, was used as positive control in all amplification reactions. Parasites were cultured and maintained *in vitro* in Liver Infusion Tryptose (LIT) [26] and DNA extracted from ∼10^4^ parasites using the same techniques described above. Once DNA was isolated, the pellet was resuspended in 8 mM NaOH at a final concentration of 10 ng/µL.

Midguts and/or fecal material were excised from EtOH-preserved whole insects and maintained in 70% EtOH until processing and *T. pallidipennis* hosts (i.e. bloodmeal sources) were identified using PCR. A conserved consensus sequence of cytochrome b was generated by alignment of sequences available for all mammal genera present at the site [25]. It is important to note that this sequence also amplifies from bird and reptile genomes, using the same primers, providing a broad range of potential host identification. Primers designed to amplify this consensus region are: DC-cytb-UP, 5-′CRT GAG GMC AAA TAT CHT TYT-3′ and DC-cytb-DW, 5′-ART ATC ATT CWG GTT TAA TRT-3′, which produced an amplified product of 420 bp. Given broad divergence in the homologous region between animal species and that reported in humans, a third primer (anti-sense) was designed to combine with the DC-cytb-UP primer, which amplifies a shorter region only of human cyt b (315 bp): H-cytb-DW 5′-AGG AGA GAA GGA AGA GAA GT-3′ (this latter sequence was incorrectly reported in [25]). The multiplex PCR reaction uses the three primers in a final volume of 12–15 µL in 1× Master Mix, with 1 µL DNA of the sample, 0.5 µM of mammal oligonucleotides (DC-cytb-UP and DC-cyt*b*-DW) and 0.25 µM human primer (H-cytb-DW 5′). The amplification reaction uses an initial denaturalization cycle (94°C for 4 min), followed by 35 cycles of denaturing (94°C, 30 sec), alignment (42.5°C, 30 sec), and extension (72°C, 30 sec), ending with a final extension period (72°C, 10 min). Amplified products were separated and visualized on 1% agarose gels under UV light. The bands corresponding to 420 bp (i.e., nonhuman mammals) were purified using Wizard® SV Gel and PCR Clean-Up System (Promega), and resuspended in 15 µL H_2_O. PCR products were sequenced in a Genetic Analyzer ABI Prism 3100 (Applied Biosystems, CA USA), using a Big Dye Terminator v3.1 cyclic sequence kit. Sequences were aligned and edited using BioEdit [27] and species identification of bloodmeals compared using BLASTN and cytochrome b mammal sequences from Genbank [28].

### Data analysis

The Chalcatzingo landscape studied herein has no apparent barriers impeding species' movements across habitats, although clear differences exist in their structure and biotic composition. We use community similarity and a combination of alpha and beta diversity analyses as measures of ecological connectivity. Fisher's alpha parameter for a fitted logarithmic series distribution was used as the diversity index among species groups, since relatively low numbers of species and individuals were present in samples [29]. All similarity and diversity indices were calculated using EstimateS v. 8.00 [30]. The Jaccard similarity index, as defined by Chao [31], was used for comparisons between adjacent habitats [32].

Statistical analyses were conducted to measure association of *T. cruzi* prevalence and body condition for rodent reservoirs, based on a median test of the body mass index (BMI; residuals of the linear regression of body mass and body length). Frequency analyses were conducted to test for *T. cruzi* prevalence biases related to sex or age of reservoirs, and odds ratios were used to determine whether any habitat, season, or species had higher *T. cruzi* prevalence than expected by chance. Association between mammal species' relative abundance and their parasite prevalence was analyzed using Pearson's correlation coefficients.

The sensitivity (proportion of positives correctly diagnosed as positive), specificity (proportion of negatives correctly identified as negative), positive predictive value (ratio of true positives from combined true and false positives) and the negative predictive value (true negative test from combined true and false negatives) were measured for bug *T. cruzi* infection using microscopy vs. molecular techniques.

Since many ecological and biological factors (i. e. relative abundance, attractiveness to vectors, infection competence) may influence the relative importance of vector host species as *T. cruzi* sources for human infections across a landscape (based on bloodmeal sources), we developed a simple index to rank host community species (including potential reservoirs). This Chagas reservoir index (CRI) is composed of the relative abundance (RA) of host species *i*, *T. cruzi* relative infection for species *i* (RI), the proportion of mixed human and species *i* bloodmeals in bugs (PMBM), and the probability of species *i* being a blood source for *T. pallidipennis* (relative prevalence of species *i* bloodmeals among all bug bloodmeals, PBM), calculated as:
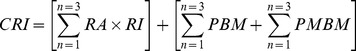



We use the relative infection per species (the number of infected individuals per species over the number of total infected individuals in each habitat per season) instead of prevalence, because the former is a better indicator of vector-host interactions in the overall community.

## Results

### Triatomine and mammal surveys

In all, 185 *T. pallidipennis* and two *T. barberi* individuals were collected in this study; the latter species is not included in our analyses, however, since no bloodmeals were obtained from either specimen. *Triatoma pallidipennis* collection success was highest in domestic habitats in the rainy season, with a non-significant decrease during the dry season though the collection method for the domestic habitat was direct, as compared with indirect (light traps) for the ecotone and sylvatic areas (Table 1). Collection success methods in sylvatic and ecotone habitats were not biased for season; collection success was lowest in the ecotone, with significantly more collected during the rainy season, in contrast to higher collections in the dry season in sylvatic habitats. *Trypanosoma cruzi* infection in bugs was relatively constant between seasons, and at least for adults, with prevalences across habitats ranging 63.6–71.4%.

**Table 1:** *Triatoma pallidipennis* collection success (number of individuals), and *Trypanosoma cruzi* infection in adult and nymph stages, from sylvatic, ecotone, and domestic habitats, during rainy and dry seasons in Chalcatzingo.

Habitat	Collection success [n]		*Trypanosoma cruzi* infection % [positives/n]					
			Season		Stages			
	Rainy	Dry	Rainy	Dry	Female	Male	Nymph IV–V	Nymph I–III
Sylvatic	0.21* [24]	0.28* [33]	71.4 [15/21]	66.7 [18/27]	72.2	78.9	40	-
Ecotone	0.14* [17]	0.08* [10]	61.5 [8/13]	66.7 [6/9]	66.7	83.3	50	25
Domestic	4.8^a^ [84]	3.6^a^ [17]	67.1 [49/73]	63.6 [7/11]	81.8	62.5	64	57.1

* Number of bugs/hr/m^2^.

a number of bugs/hr/house.

Twelve bat species were collected accross sampling seasons (Table 2). Species common to sylvatic and ecotone habitats in the rainy season were principally frugivorous and insectivorous: *Artibeus lituratus*, *Sturnira lilium* and *Pteronotus parnelli*. *Artibeus jamaicensis* was the only species trapped in all three habitats in at least one season. *Desmodus rotundus* was the only species collected from both sylvatic and ecotone habitats in the dry season.

**Table 2:** Bat species collected from caves and open areas in the vicinity of Chalcatzingo in rainy and dry seasons: S = sylvatic, E = ecotone, D = domestic areas.

Family	Species	Rainy			Dry			Capture site	Guild
		S	E	D	S	E	D		
Emballonouridae	*Balantiopterix plicata*	50	-	-	6	-	-	c	i
Mormoopidae	*Pteronotus parnelli*	1	1	-	6	-	-	c	i
Phyllostomidae	*Artibeus jamaicensis*	3	-	2	-	1	3	o	f
	*Artibeus lituratus*	-	5	1	-	1	3	o	f
	*Choeronycteris mexicana*	-	2	-	-	-	1	o	nf
	*Dermanura* sp.	1	-	-	-	-	-	o	f
	*Desmodus rotundus*	-	5	-	2	9	-	o	s
	*Glossophaga soricina*	-	-	-	2	-	-	o	nf
	*Leptonycteris yerbabuenae*	-	2	-	-	-	-	o	nf
	*Macrotus waterhousii*	5	-	-	1	-	-	o	i
	*Micronycteris megalotis*	-	1	-	-	-	-	o	i
	*Sturnira lilium*	1	1	-	-	-	-	o	f

Capture site: c = cave entrance, o = open habitat. Guild: f = frugivorous, i = insectivorous, n = nectarivorous, s: sanguinivorous.

Seven rodent species were collected over rainy and/or dry seasons, from at least one of the three habitats. Over half of all rodent species were present in all habitats in the rainy season (*Rattus rattus*, *Baiomys musculus*, *Peromyscus levipes*, *Sigmodon hispidus*; Table 3). Two species were not found in domestic habitats in any season (*Neotoma mexicana* and *Liomys irroratus*) and *Mus musculus* was never detected in sylvatic habitats. These latter three species are considered modified habitat restricted species, while *R. rattus*, *B. musculus*, *P. levipes*, and *S. hispidus* are considered habitat non-restricted species, since they were present in all three habitats, at least in the rainy season; the latter two were collected in all three habitats in both seasons (Table 3). *Liomys irroratus* and *S. hispidus* were the most abundant rodents in the sylvatic habitat in the rainy and dry seasons, respectively; *S. hispidus* was also the most abundant species in the ecotone, while *M. musculus* was the most abundant domestic rodent species in both seasons.

**Table 3:** Rodent species collected and their infection with *Trypanosoma cruzi* (Inf) from sylvatic, ecotone, and domestic habitats over rainy and dry seasons in the vicinity of Chalcatzingo, Morelos, Mexico; (N) = total individuals collected, (CI) = Confidence Intervals).

Species	Within each species												Among species	
	Rainy						Dry							
	Sylvatic		Ecotone		Domestic		Sylvatic		Ecotone		Domestic			
	Inf (N)	OR	Inf (N)	OR	Inf (N)	OR	Inf (N)	OR	Inf (N)	OR	Inf (N)	OR	Inf (N)	OR
		(CI)		(CI)		(CI)		(CI)		(CI)		(CI)		(CI)
Restricted														
*L.irroratus*	3 (40)	1.7	1 (10)	1.8	(NC)	N/A	0 (11)	N/A	0 (1)	N/A	(NC)	N/A	4 (62)	0.5
		(0.2–17.4)		(0.2–19.5)										(0.2–1.5)
*M. musculus*	-	N/A	1 (6)	3.7	3 (38)	2.2	(NC)	N/A	(NC)	N/A	0 (21)	N/A	4 (65)	0.5
				(0.3–42.9)		(0.2–22.7)								(0.2–1.4)
*N. mexicana*	0 (6)	N/A	0 (1)	N/A	(NC)	N/A	5 (17)	N/A	(NC)	N/A	(NC)	N/A	5 (19)	**3**
														**(1.02–9)** *
Non-restricted														
*B. musculus*	0 (2)	N/A	3 (8)	1.8	1 (4)	0.9	2 (8)	0.9	2 (8)	0.9	-	N/A	8 (30)	**3.5**
				(0.31–10.4)		(0.1–10.2)		(0.1–5.7)		(0.1–5.7)				**(1.4–8.5)** *
*P. levipes*	1 (2)	11	0 (7)	N/A	1 (8)	1.3	2 (17)	1.3	0 (3)	N/A	0 (1)	N/A	4 (40)	0.9
		(0.5–223.9)				(0.1–14.3)		(0.2–10.1)						(0.3–2.6)
*R. rattus*	0 (1)	N/A	0 (1)	N/A	2 (4)	N/A	-	N/A	-	N/A	0 (1)	N/A	2 (7)	3.3
														(0.6–17.8)
*S. hispidus*	0 (5)	N/A	2 (27)	0.8	1 (2)	11.8	5 (34)	3.1	0 (19)	N/A	0 (4)	N/A	8 (91)	0.8
				(0.1–4.1)		(0.7–208)		(0.7–13.9)						(0.3–1.8)

Species are grouped according to ecological preferences (i.e. restricted specifically to any one habitat or not). Odds ratios (OR) were calculated as the proportion of infected individuals within each collection category as compared with all others within the species, or of the combined infection of each species among all other species; OR <1 represents less infection rate than expected and >1 as greater infection rate than expected by chance. (NC = not collected; N/A = not applicable;

* = significance at the 95% confidence interval).

### Rodent community

Fisher's alpha diversity indices were similar between seasons and habitats, albeit with a consistent but non-significant trend toward lower diversity in the dry season. Rodent community similarity (1-beta diversity), however, was affected by habitat and season (Table 4). High similarity values were observed between sylvan and ecotone habitats, while lower values were observed between domestic and sylvan habitats, in both seasons. In contrast, significantly lower similarity values were observed between domestic and ecotone in the dry as opposed to the rainy season. Only one rodent species, the cotton rat *S. hispidus*, was collected in all three habitats and both seasons.

**Table 4:** Rodent community similarity indices based on the Chao-Jaccard index (standard deviation), between habitats for dry (above diagonal) and rainy (below diagonal) seasons.

	Sylvatic	Ecotone	Domestic
Sylvatic	-	0.79 (0.24)	0.17 (0.28)
Ecotone	0.91 (0.11)	-	0.18 (0.28)
Domestic	0.11 (0.19)	0.82 (0.18)	-

### *Trypanosoma cruzi* mammal reservoirs

None of the 116 bats collected in this study was infected with *T. cruzi*, and hence, assuming no sample size bias, bats were significantly less infected than expected, as compared with rodents (Wilson score [33] for intervals at 95% = 0–0.039 for bats, in contrast to 0.09–0.17 for rodents).

**Figure 2:**
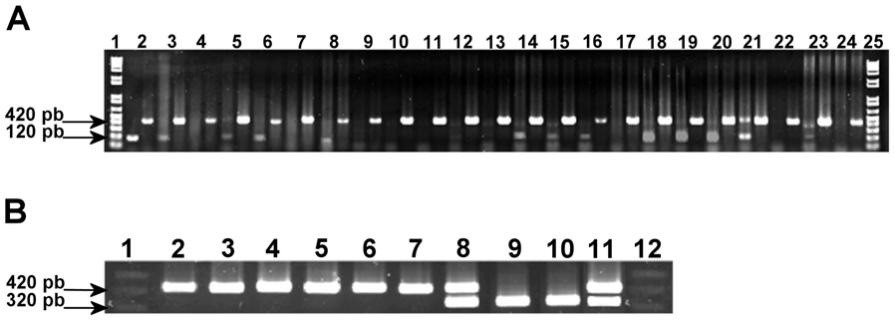
PCR diagnosis of *Trypanosoma cruzi* in mammals and *T. pallidipennis*, and bug bloodmeal content. *(* ***A*** *)* Identification of *Trypanosoma cruzi* (120 bp) and amplification of animal cytochrome b (420 bp) from rodent specimens as DNA positive control: (3–5) *Neotoma mexicana*, (6–8) *Sigmodon hispidus*, (9–16) *Baiomys musculus*, (17–19) *Mus musculus*, (20) *Rattus rattus*, (21, 22) *Liomys irruratus*, (23, 24) *Peromyscus levipes*. Lanes 2, 3, 5, 6, 8, 14, 15, 16, 18, 19, 20, 21, and 23 were positive for *Trypanosoma cruzi*. *(* ***B*** *) Triatoma pallidipennis* bloodmeal identification using cytochrome b sequencing. Human blood amplified a 320 bp sequence (lanes 8 to 11), while all animal blood amplified a 420 bp sequence (lanes 2 to 8 and 11). Animal sequences amplified were later sequenced as *Didelphis virginiana* from an adult female (lane 2), *Canis familiaris* from a stage 2 nymph (lane 3), *Felis catus* from a stage 5 nymph (lane 4-), *Gallus gallus* from a male (lane 5), *Sigmodon hispidus* from a female (lane 6), *Mus musculus* from a stage 5 nymph (lane 7), *S. hispidus* and human from a stage 3 nymph (lane 8), Human from a female (lane 9), human from a stage 5 nymph (lane 10), *M. musculus* and human from a female (lane 11). PCR conditions allowed double bloodmeal amplification (lane 8 and 11). Lanes 1 and 12 are molecular weight markers.

At least one individual from each rodent species was positive for *T. cruzi* in cardiac tissue (Table 3, Figure 2A). Only *T. cruzi* lineage I was identified from positive mammal samples (data not shown). Infection rates for individual reservoir species varied from 6.2% (*M. musculus*) to 28.6%. *Neotoma mexicana*, and *B. musculus*, had the highest infection rates, (Table 3). There was an association between sample size and *T. cruzi* detection capacity in niche restricted species, which was not observed for non-restricted species. Seasonal differences in *T. cruzi* prevalence were observed in *S. hispidus* associated with ecotone/sylvatic habitat use, whereas infected *B. musculus* showed a persistent use of ecotone while expanding to the domestic habitat in the rainy season. *Trypanosoma cruzi*-infected rodents had a significantly greater body mass, as compared to non-infected individuals (median test = 24, Z-score = 2.35, DF = 1, P = 0.019). Neither sex (two-tailed chi-square = 0.025; 1 d.f.; P = 0.88), nor age (two tailed chi square = 1.52; 1 d.f.; P = 0.22) were associated with *T. cruzi* infection prevalence in rodents.

**Figure 3:**
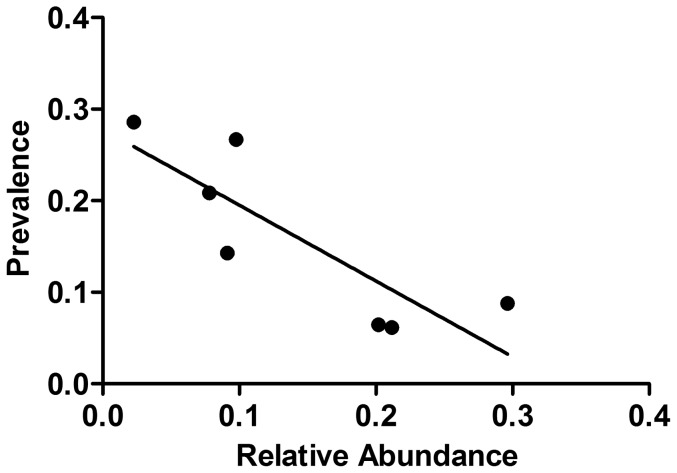
Correlation analysis between rodent relative abundance (proportional abundance of each species), and *Trypanosoma cruzi* prevalence (proportion of infected individuals of each species).

**Figure 4:**
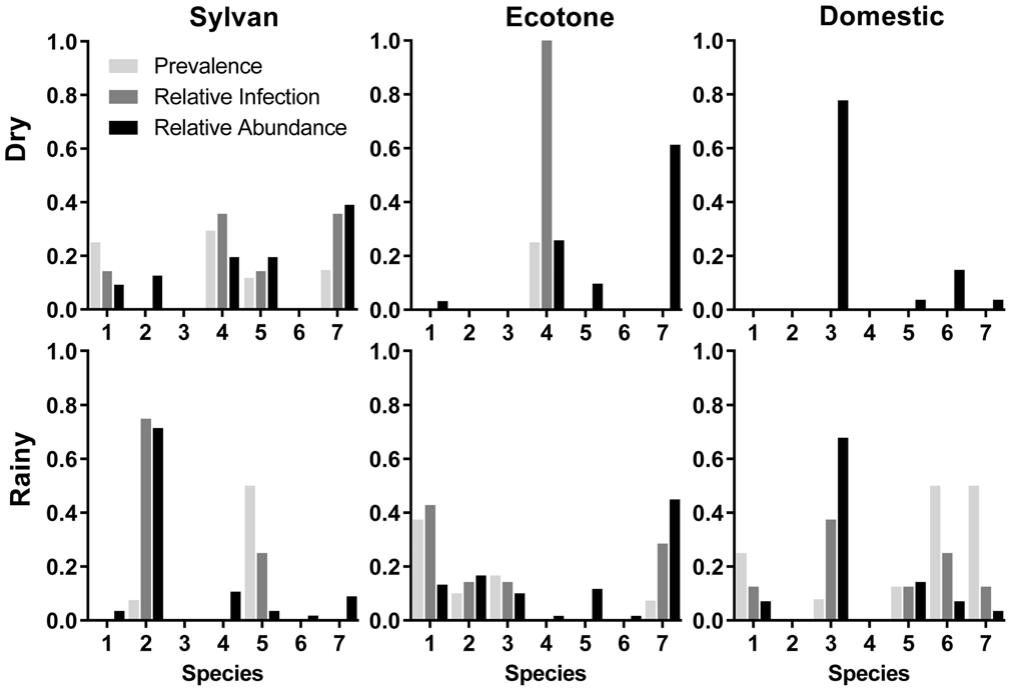
Association between reservoir species' relative abundance and their relative *Trypanosoma cruzi* infection rates and prevalence for (1) *Baiomys musculus*, (2) *Liomys irroratus*, (3) *Mus musculus*, (4) *Neotoma mexicana*, (5) *Peromyscus levipes*, (6) *Rattus rattus*, and (7) *Sigmodon hispidus*, over both seasons and in all three habitat types.

*Trypanosoma cruzi* prevalence in rodent species was negatively correlated with species' relative abundances (Pearson correlation = −0.84, P = 0.018, Figure 3). However, each species' *T. cruzi* prevalence was not associated with individual species' relative *T. cruzi* infection, based on the complete rodent community, as would have been expected for a non-specialist parasite (Pearson correlation = −0.05, P = 0.92, Figure 4). Although quantitative analysis of *T. cruzi* relative infection and abundance was not possible for most individual species due to low or nil sample collections across all habitats, there was an important qualitative pattern difference among habitats for most species. Neither season nor habitat type, were associated with *T. cruzi* infection at the landscape level. However, *T. cruzi* infection for all rodents combined was significantly higher in the dry season in the sylvan habitat (Table 5, OR = 5.37, 95%CI = 1.17–12.77). Specifically, sylvan *N. mexicana* had significantly higher *T. cruzi* infection in the dry season, as compared with the other three species present.

**Table 5:** Association of *Trypanosoma cruzi* infection with season, habitat and species.

All rodents/Habitat/Season Level	Odds Ratio	CI 95%
Seasons (landscape level)		
Rainy/Dry	1.001	0.5–2.03
Habitat (combined seasons)		
Sylvatic/D+E	1.42	0.71–2.84
Ecotone/D+S	0.84	0.38–1.88
Domestic/E+S	0.82	0.36–1.88
Habitat/Rainy		
Sylvatic/D+E	0.52	0.16–1.64
Ecotone/D+S	1.1	0.41–2.96
Domestic/E+S	1.6	0.6–4.21
Habitat/Dry		
Sylvatic/D+E	5.37	1.17–24.6*
Ecotone/D+S	0.43	0.11–2.29
Domestic/E+S	N/A	
Species Level for Sylvatic/Dry		
*Neotoma mexicana*/Sh+Bm+Pl	3.79	1.124–12.77*
Sigmodon hispidus/Nm+Bm+Pl	1.57	0.5–4.9
Baiomys musculus/Nm+Sh+Pl	2.93	0.54–15.9
Peromyscus levipes/Nm+Sh+Bm	1.09	0.22–5.25

(D = domestic, E = ecotone, S = sylvatic). (* = Significant departure from random expectation at 95% of confidence).

### Triatomine infection

**Figure 5:**
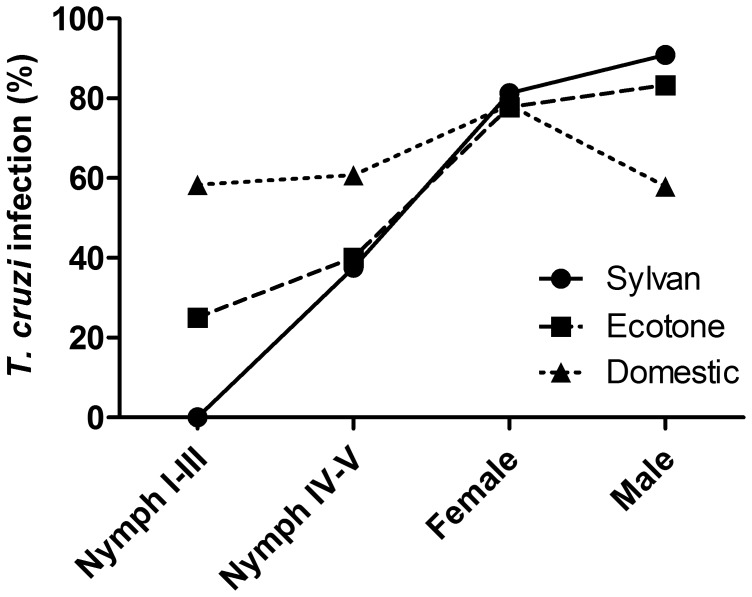
*Trypanosoma cruzi* infection rates in *Triatoma pallidipennis* nymphs and adults collected from sylvatic, ecotone, and domestic habitats in Chalcatzingo.

The detection capacity of *T. cruzi* in triatomines using microscopy was similar to that using PCR. The sensitivity of microscopy as compared to PCR was independent of season, with 90.9% specificity and a 95.5% positive predictive value. However, the negative predictive value for microscopy was 58.8%. All triatomine *T. cruzi* infection values reported herein were analyzed using PCR (Table 1); all samples isolated from *T. pallidipennis* in this study were identified as *T. cruzi* lineage I (data not shown). There was a marked difference among habitats in bug infection rates for juveniles, even though there was no seasonal difference in overall bug infection rates among habitats. Early stage nymphs (I–III) from the domestic habitat had significantly higher infection rates than those from ecotone or sylvatic areas. In addition, females had double the infection rate as compared with males and nymphs in the domestic habitat (Table 1, Figure 5).

### Triatomine hosts

Of the 185 bugs collected, 145 foregut samples successfully amplified for *T. cruzi* primers, while only 57 samples amplified using cyt b primers for host identifications (Table 6, Figure 2B). Both bloodmeal type and *T. cruzi* infection were identified from a total of 42 rainy season and 15 dry season bugs. Blood from six non-human species was identified from bug bloodmeals: *Sigmodon hispidus*, *Mus musculus*, *Didelphis virginiana*, *Canis familiaris*, *Felis catus*, and *Gallus gallus*.

**Table 6:** Bloodmeal identification of bug foregut contents and *Trypanosoma cruzi* infection prevalence.

Species	Rainy season								Dry season							
	All habitats		Sylvatic		Ecotone		Domestic		All habitats		Sylvatic		Ecotone		Domestic	
	[107]	N	[15]	N	[25]	N	[67]	N	[38]	N	[10]	N	[22]	N	[6]	N
*Sigmodon hispidus*	100.00%	3		0	100.00%	2	100.00%	1	100.00%	2		0	100.00%	2		0
*Mus musculus*	80.00%	5		0	66.70%	3	100.00%	2	100.00%	2		0	100.00%	2		0
*Didelphis virginiana*	66.70%	3	100.00%	1	0.00%	1	100.00%	1	100.00%	1		0	100.00%	1		0
*Canis familiaris*	100.00%	3	100.00%	1	100.00%	1	100.00%	1	100.00%	1		0	100.00%	1		0
*Felis catus*	50.00%	4		0	0.00%	1	66.70%	3		0		0		0		0
*Gallus gallus*	100.00%	1		0		0	100.00%	1		0		0		0		0
Degraded	47.70%	65	63.60%	11	63.60%	11	39.50%	43	56.50%	23	50.00%	8	54.50%	11	75.00%	4
Human	54.50%	11		0	33.30%	6	80.00%	5	100.00%	9	100.00%	2	100.00%	5	100.00%	2
Only animal	77.40%	31	100.00%	4	62.50%	8	78.90%	19	83.30%	6		0	83.30%	6		0
Human+animal	50.00%	10		0	33.30%	6	75.00%	4	100.00%	3		0	100.00%	3		0
Only human	100.00%	1		0		0	100.00%	1	100.00%	6	100.00%	2	100.00%	2	100.00%	2

Bugs were collected in rainy and dry seasons, from sylvatic, ecotone, and domestic habitats. N = total insects analyzed.

Bloodmeal rates were similar between seasons for most non-human animal species (Table 6), but human bloodmeals were significantly more frequent in the dry season in all habitats, either alone or in combination with another blood source. Double bloodmeals having both human and other animal sources, were similarly prevalent in both seasons (8.5% rainy and 7.9% dry), while bloodmeals containing only non-human animal blood were almost twice as prevalent in the rainy season (26.5% vs. 15.8%). Non-human animals from double bloodmeals were *S. hispidus*, *M. musculus*, or *D. virginiana*. *Gallus gallus* was identified only from triatomines collected in domestic areas, while *C. familiaris* and *F. catus* were a blood source in both ecotone and domestic habitats. Blood from *M. musculus* was identified from domestic and ecotone bugs in both seasons.

Although 145 bug foreguts contained non-degraded DNA for bloodmeal analysis, based on amplification of *T. cruzi* primers and DNA controls, 60.7% of these could not amplify using consensus non-arthropod (for mammal-avian-reptile taxa) cytochrome b primers. A significantly higher rate of these samples in the dry season was infected.

### Reservoir Index

Eleven species were ranked according to an index (CRI) constructed using four components of *T. cruzi* reservoir importance which model and measure vertebrate-parasite and vertebrate-vector interactions (Table 7). The three most important non-human animal species associated with bugs having human bloodmeals were *M. musculus* (niche-restricted rodent), *S. hispidus* (a niche non-restricted species) and *D. virginiana*, another non-restricted species. These species were more frequently associated with *T. cruzi* infection and *T. pallidipennis* bloodmeals, at the same time. The chicken (*G. gallus*) is included in the analysis, not because this is a *T. cruzi* reservoir, but because it is a blood source for infected triatomines.

**Table 7:** Chagas reservoir index (CRI), which measures the relative importance of *Triatoma pallidipennis* hosts as competent reservoirs for *T. cruzi* associated with human infection.

Species	RI * RA	PBM	PMBM	CRI	Rank
*Mus musculus*	0.13	0.36	0.57	1.06	1
*Sigmodon hispidus*	0.14	0.2	0.14	0.48	2
*Didelphis virginiana*	0.02	0.16	0.14	0.32	3
*Liomys irroratus*	0.28	0	0	0.28	4
*Canis familiaris*	0.02	0.16	0	0.18	5
*Felis catus*	0.01	0.16	0	0.17	6
*Neotoma mexicana*	0.16	0	0	0.16	7
*Gallus gallus* *	0	0.04	0	0.04	8
*Baiomys musculus*	0.04	0	0	0.04	9
*Peromyscus levipes*	0.03	0	0	0.03	10
*Rattus rattus*	0.01	0	0	0.01	11

* *T. pallidipennis* host but not a *T.cruzi* host.

## Discussion

This study aimed to characterize all habitat types where local human populations could be exposed to and potentially interact with triatomine bugs, as well as to characterize and quantify the parasite's interactions within an epidemiologically relevant Mexican Chagas disease transmission landscape. Previous livestock and human population seroprevalence and *T. pallidipennis* population dynamics studies [14]–[16] had pointed directly to diverse sources of reinfesting domestic bugs following control interventions, with primary infestation risk factors associated with the presence of wild, livestock, and domestic animals in and around houses, in addition to multiple sociocultural components (occupation, cultural practices, livestock confinement practices, household economy and priorities). An integrated approach to Chagas disease prevention and control implies understanding the parasite sources in the complete landscape where humans are exposed, interact, and modify directly or indirectly host/reservoir community. The present study provides primary evidence using ecological parameters, for differential potential for parasite population flow among habitats and according to seasons.

We observed high species richness of potential *T. cruzi* reservoirs and *T. pallidipennis* hosts of terrestrial and flying small mammals in both sylvatic and modified habitats in this study. Chalcatzingo, located within the Mexican Transvolcanic Belt, is well known for high mammal diversity, despite expansion of modified habitats [34]. The distinct habitats of the Chalcatzingo landscape are nonetheless ecologically connected via biotic interactions associated with *T. cruzi* transmission. However, connectivity was variable over seasons, as evidenced by the reduced similarity between habitats in the dry season. Although the same vector is present in all habitats, only a few rodent species actually use the entire landscape; those that do are known to be important agricultural pests [35]. One of the most important agricultural pests in Mexico, *Sigmodon hispidus*, feeds on seeds during the growing season (rainy season), and disperses into sylvatic habitats during the dry season [36]. This species provides at least one route for *T. cruzi* dispersal via vectors, present and feeding in all habitats.

None of the bat specimens collected in this study was positive for *T. cruzi* infection even though *Artibeus jamaicensis* and *A. lituratus* roosting sites located in rock outcrops in sylvatic habitats in Morelos and surrounding Chalcatzingo are infested with *T. pallidipennis* and can have high *T. cruzi* prevalences (90%, Ramsey unpublished data). Bat species have often been implicated in transmission cycles of *T. cruzi*, both as hosts and predators of Triatominae [37]. The absence of *T. cruzi* in our bat samples may be the result of few collections for the majority of species, and the fact that abundant species have typically large roosting populations, since other studies have found low *T. cruzi* prevalence in bats, including D'Alessandro and Barreto [38], who report <9% infection in 3709 individuals examined. *Artibeus jamaicensis* has been found infected with *T. cruzi* in Colombia [39], Brazil [40], and Argentina [41], and in the southern Mexican states of Chiapas and Campeche where the *dimidiata* complex species are present (Ramsey et al., unpublished data). This bat species is commonly found in towns or dwellings [42], where they roost or feed on *Ficus* or *Ceiba* in public parks. Tree holes of both species are documented to be used as refuges by triatomines of the *phyllosoma* complex [43].

In contrast to bats, all rodent species were infected with *T. cruzi* in this study, albeit with differing prevalences and hence, none of the habitats was isolated in terms of *T. cruzi* infection in rodents. Parasite prevalence in rodents was inversely related to each species' relative abundance, as has been reported for other pathogen-host systems [44]. A previously proposed mechanism to explain such a pattern is the juvenile dilution effect, which suggests that the probability of chronic infection for a given individual host increases with age [45], [46]. However, our results do not support this hypothesis, since we did not find higher infection prevalence in adult rodents, as compared with juveniles. Abundant species such as *S. hispidus*, *L. irroratus*, *M. musculus*, and *P. levipes* had lower infection rates than did less abundant species, such as *B. musculus* and *R. rattus* (prevalence rates close to 30%). *Rattus rattus*, a common inhabitant of domestic environments, has been reported previously to have high *T. cruzi* infection rates [41]. *Sigmodon hispidus*, *B. musculus*, *N. mexicana*, *P. levipes*, and *L. irroratus* have been previously found infected with *T. cruzi* in sylvatic and ecotone habitats in and around Santa Cruz Papalutla, Oaxaca, and several sites in Jalisco (Ramsey et al., in preparation), confirming that they are probably important *T. cruzi* reservoirs across a broad geographic range.

The primary triatomine species in Chalcatzingo, based on abundance, was *T. pallidipennis*, which was collected at virtually all stages of development, in all habitats and in both seasons showing that this species is resident and reproductive year-round in all habitats [16]. These findings confirm previous observations that *T. pallidipennis* is a generalist, inhabiting diverse conserved and modified habitats, and with high ability for domestication, in west-central Mexico [47], [48]. *Trypanosoma cruzi* prevalence in *T. pallidipennis* in the present study was consistent with observations over 10 yrs of study in the community, and was high in all habitats, in both seasons, and particularly for early developmental stages in the domestic habitat. Together with high prevalence of infection in non-identifiable avian-mammal-reptile bloodmeals, and the higher crowding indices especially in the domestic habitat, these data suggest coprophilia as an additional potential source of *T. cruzi* infection for triatomines.

*Triatoma pallidipennis* was never identified with human blood from sylvatic habitats in the rainy season, but does use this source in that habitat in the dry season, alone or in combination with non-human sources. This trend supports the evidence that *T. pallidipennis* is an opportunist, feeding on diverse mammals, depending on availability [25], [47]. *Sigmodon* and *Didelphis* were consistent blood sources across habitats in the rainy season, but were absent from domestic and ecotone bloodmeals in the dry season, when no mixed human and animal bloodmeals were identified. Rainy season domestic bloodmeals contained species not recorded from the dry season, while shared human bloodmeal sources contained blood from *D. marsupialis*, *F. catus*, and *C. familiaris*, in domestic and ecotone habitats. The lack of sufficient non-human blood sources, probably owing to decreased rodent abundance in the dry season, provides the opportunity for human bloodmeals, and the potential for parasite transmission.

Agricultural and urban rodent pests were the most relevant species associated with human *T. cruzi* exposure hazard in this *T. pallidipennis*-infested landscape. Although the CRI was calculated based on relatively low sample sizes, the results provide evidence of reservoir, vector, and parasite interactions across the landscape. Rodent pests are responsible for outbreaks of some of the more important rodent-borne zoonoses around the world [49], [50], such as the white-footed mouse *Peromyscus leucopus*, a reservoir of *Borrelia burgdorferi*, the pathogen causing Lyme disease and transmitted by ticks [51].

The first complete community triatomine control trial in Mexico was conducted in Chalcatzingo in 1999: following three insecticide spray rounds, the latter also including rodent control within the community, >95% of domestic *T. pallidipennis* infestation was eliminated. However, neither county nor state healthcare officials continued these interventions, and by 5 yr post interventions, infestation rates had recovered to pre-trial levels [Ramsey, personal communication]. As demonstrated in the present study, *T. pallidipennis* maintains high infestation and *T. cruzi* infection rates in spatially and biotic-interconnected habitats, providing ample sources for domestic reinfestations, particularly in the rainy season.

This study provides a framework for development of new and more effective integrated pest management strategies, based on the spatio-temporal variation of reservoirs and triatomines along the landscape matrix of sylvatic, ecotone, and domestic habitats. Application of triatomine control would be most efficient in the dry season, when lack of available food sources forces triatomines to forage and contact human population, and potentially insecticides (Ramsey, personal communication). Inhabitants of Chacatzingo and other communities infested with *T. pallidipennis* commonly report sighting more triatomines inside houses in the late dry season (March–May), which provides the opportunity for Chagas prevention activities to target interventions integrating biological and ethnographic/social components more effectively.

Correlatively, the present study provides evidence for increased bug-human interactions in the dry season, the same season when domestic reservoir communities are less continuous with the other habitats. While primary acquisition of parasites by triatomines may occur in the rainy season through multiple sources in all habitats, connectivity of vector populations via key mammal sources, such as humans, occurs principally in the dry season. Hence, focusing control activities on dry season hosts may decrease bug population survival and fitness, or force bugs to search for other hosts, alternatives that need to be considered before strategies are implemented. Reduction of *T. cruzi*-infected rodent reservoir populations would also have to target principal agricultural pests (*S. hispidus*, *P. levipes*, and *B. musculus*) [51]. A joint, integrated pest management program between health and agriculture ministries would thus offer double benefits, being cost-effective control for both public health and farming economies.

Most national regulatory norms for Chagas disease continue to include investment in the training, equipment and inclusion of light microscopy to detect and monitor domestic bug infection. Unfortunately, less than 10% of bugs reach health services alive, and based on the low negative predictive value for microscopy measured in this study, this method may represent a cost-inefficient method to opportunely diagnose presence or absence of the parasite in vector populations. Independent of whether a bug is infected or not, primary healthcare personnel should carry out a household interview, and take blood samples for diagnosis for all exposed individuals if pertinent. Information on bug infectivity with such high negative predictive value would be ineffective to measure transmission risk or make vector control decisions. Conducting periodic surveys (every 1–3 yrs) in sentinel sites, with representative sampling across landscapes, and monitoring infection rates using PCR analysis would be more effective and cost-efficient, especially if an active population-based surveillance and vector control program exists in the community.

Ecological connectivity of sylvatic, ecotone, and domestic habitats provides opportunities for reservoir species dispersal. Data generated using ecological parameters such as in this study, need to be complemented using parasite, vector and potentially, mammal population genetics. This point is relevant not only for *T. cruzi* transmission, but also for other zoonotic diseases such as leishmaniasis [41], hantavirus [42], leptospirosis [44], and others yet to be fully documented in Mexico. As long as habitat modifications continue, urban areas grow, biotic communities and their interactions between organisms are modified, human populations will be exposed via different and evolving routes to zoonotic infectious agents. Early detection and surveillance of this exposure will require analysis of landscapes and all organisms interacting with pathogen transmission, human social and cultural parameters, as well as the community's awareness and participation with surveillance and prevention strategies.
